# Neighborhood Attributes and Well-Being Among Older Adults in Urban Areas: A Mixed-Methods Systematic Review

**DOI:** 10.1177/0164027521999980

**Published:** 2021-04-28

**Authors:** Miguel Padeiro, José de São José, Carla Amado, Liliana Sousa, Carla Roma Oliveira, Alina Esteves, Jennifer McGarrigle

**Affiliations:** 1 450735 CEGOT (Centre of Studies in Geography and Spatial Planning), Department of Geography and Tourism, University of Coimbra, Coimbra, Portugal; 2 Interdisciplinary Centre of Social Sciences (CICS.NOVA), Faculty of Social Sciences and Humanities (NOVA FCSH) & Faculty of Economics, 70985 University of Algarve, Portugal; 3 Center for Advanced Studies in Management and Economics (CEFAGE) & Faculty of Economics, 70985 University of Algarve, Portugal; 4 Center for Health Technology and Services Research, 56062 University of Aveiro, Portugal; 5 386368 Institute of Geography and Spatial Planning, Universidade de Lisboa, Lisbon, Portugal

**Keywords:** aging, neighborhood, environment, well-being, older adults, urban areas

## Abstract

Expanding urbanization rates have engendered increasing research examining linkages between urban environments and older adults’ well-being. This mixed-methods systematic review synthesizes the evidence for the influence of urban neighborhoods’ attributes on older adults’ well-being. We searched for literature published up to December 2020 across six databases and performed quality assessment and thematic analysis. The results, based on 39 identified studies, showed that natural areas in neighborhoods and a sense of community are the attributes most often associated with positive effects on well-being. Transit-related variables, urban furniture, and access to healthcare are also positively related to well-being. Neighborhoods may promote well-being more effectively when these elements are considered. However, almost half of the studies did not include all environmental dimensions simultaneously, and self-reported instruments were largely preferred over more objective assessments of the environment. Future research should thus holistically examine physical, social, and service-related attributes to produce more robust evidence.

## Introduction

In a world where more than half of the population now lives in urban areas (UNDESA, 2019) and with the proportion of people aged 65+ expected to double to 1.5 billion by 2050 (UNDESA, 2020), population aging and urbanization are now key interrelated topics. Between 1990 and 2015, the number of people aged 65+ living in urban areas rose from 160 to 355 million globally, representing a growth of 122% (UNDESA, 2014). Currently, 58.8% of people aged 65+ live in urban areas, compared to 48.3% in 1990. During the same period, their number increased by 70 million (+71%) in UN-defined “more developed countries” (+13.7 million in the U.S., +31.6 million in European countries) and by 125 million (+125%) in “less developed countries” (UNDESA, 2014).

Since 2007, the World Health Organization (WHO) has emphasized the need to make our cities more age-friendly, as a necessary condition to promote older urban residents’ well-being (WHO, 2007). This has become a key priority (Dhéret et al., 2011; OECD, 2013; Tinkler & Hicks, 2011), as demonstrated by the *Global Strategy and Action Plan for Ageing and Health for 2016–2020* and the *Decade of Healthy Ageing 2020–2030* (Rudnicka et al., 2020; WHO, 2017). The definition of healthy aging adopted by the WHO highlights the importance of well-being, defined as “the process of developing and maintaining the functional ability that enables well-being in older age.” In recognizing this functional ability, as influenced by interactions between individuals’ intrinsic capacity and environmental characteristics (WHO, 2017) and by prioritizing global network enhancement for age-friendly cities and communities (WHO, 2007), the need to better understand the relationship between the outdoor residential environment and older adults’ well-being becomes clear. This mixed-methods systematic review therefore aims to synthesize the existing knowledge regarding the influence of urban neighborhood attributes on aged individuals’ well-being. This review’s findings may inform planning policies and practice by documenting which elements of the local environment are more likely to contribute to positive feelings in later life.

## Defining Well-Being

Well-being has long been an object of research in gerontology (Campbell, 1976; Larson, 1978) and a major topic in human communities and societies. The concept has been defined, operationalized, and measured in multiple ways (Dodge et al., 2012; Liu et al., 2017; Nordbakke & Schwanen, 2014; Thomas, 2009), and no single, universal definition exists. In this review we leave aside two categories of well-being: physical well-being, considered critical for public health purposes (Üstün et al., 2010), and negative psychological conditions such as stress, anxiety, and depression (Cassano & Fava, 2002). This is because we are interested in longer term based positive components of well-being, which reflect flourishing feelings and a positive outlook on life (Diener, 2000; Medvedev & Landhuis, 2018; Ryff & Keyes, 1995). The existence of health problems, of ongoing incapacitation processes, and even of negative affects is not incompatible with an overall positive assessment of life (Golant, 2015; Rubinstein & de Medeiros, 2015), particularly when the time interval considered extends (Mouratidis, 2018; Nordbakke & Schwanen, 2014).

At least four intertwined concepts relating to this broader meaning of well-being have been applied in the literature and can be included in this review: subjective well-being, happiness, satisfaction with life, and the psychological component of quality of life—as this last concept also includes objective variables assessed by the observer (Bowling et al., 2003; Meeberg, 1993), such as material resources, health status, social status, or housing conditions. They are frequently overlapped or used interchangeably (Haas, 1999; Medvedev & Landhuis, 2018), and they all involve a balance between psychological, social, and physical resources and the challenges faced (Dodge et al., 2012). Together they can provide a good indicator of an individual’s relationship to his/her life (Ní Mhaoláin et al., 2012), relying on their own assessment (Diener et al., 1999), and giving major importance to self-realization, self-fulfillment and the pursuit of meaningful goals for the individual (Nordbakke & Schwanen, 2014; Ryan et al., 2013), while not rejecting the affective (hedonic) component of well-being. For some authors the term can refer to a sense of meaning and purpose in life and to notions of autonomy, control, and achievement (Ryff & Singer, 1998; Tinkler & Hicks, 2011). For others it evokes a well fulfilled life and includes both cognitive and affective dimensions (Watson et al., 2018). For the purpose of this review, we retain the definition provided by Dodge et al. (2012, p. 230): a “stable wellbeing is when individuals have the psychological, social and physical resources they need to meet a particular psychological, social and/or physical challenge.” This dynamic and flexible definition provides aging adults with agency that reflects their own needs, meanings, representations, and expectations (Graham & Shier, 2010), and transcends relative distinctions between the different—but generally correlated with each other—constructs that contribute to overall wellness (Kahn & Juster, 2002; Medvedev & Landhuis, 2018).

## Neighborhood-Level Factors and Well-Being

The social-ecological approach (Stokols, 1992, 1996) is among the most popular frameworks for analyzing how a complex set of individual, interpersonal, and environmental factors (institutional, community/society, and policy) can affect people’s well-being. This model relies on recognition of the specificities of the local context and the existence of interactions between factors in shaping health outcomes, behaviors, well-being, and/or perceptions (Cerin et al., 2017), meaning that the effects of one environmental characteristic can be moderated by others. Contextual variables are particularly important for older people, who spend more time at home and in the surrounding environment than other age groups (Matthews et al., 2012), undertake fewer daily trips, and travel shorter distances than younger adults (Horgas et al., 1998; Rowles, 1978). Their greater reliance on local resources leaves them more sensitive to their neighborhood’s quality.

While no consensus on the precise definition of “neighborhood” exists (Kearns & Parkinson, 2001), the term denotes a socio-spatial concept that designates a portion of urban territory that is collectively recognized as a distinctive physical, functional, and social entity (Barton, 2013). The way in which a neighborhood supports its residents’ needs depends on the materiality of the local environment (the physical component), the services it provides (the functional component), and the community in which people are embedded (the social component). These components also correspond to the frequent categorization of neighborhood-level factors that may influence people’s well-being: the physical, social, and service environment (Cubbin et al., 2008; Culhane & Elo, 2005; DeLaTorre & Neal, 2017; Robert, 1999; Wen et al., 2006). These dimensions are part of a set of complex interactions that also affect individual (socio-economic situation, demographic characteristics, psychological traits) or collective (such as public policies, economic dynamics) dimensions (Menec et al., 2011; Robert, 1999).

The physical environment—frequently termed the “built” environment—has been defined as the “objective and perceived characteristics of the physical context in which people spend their time” (Van Cauwenberg et al., 2011). It generally includes various street-level features, including public space design, sidewalks, crossings, and community-level features, such as land-use characteristics, built-up densities, and the existence and accessibility of green areas (Hanson et al., 2012). A visually pleasing, neat, and walkable environment including street furniture (benches, shade) would encourage older people to leave their homes, potentially increasing social interactions and physical activity, ultimately enhancing well-being and improving (or postponing) health problems (Mouratidis, 2018; Yen et al., 2009). The service environment is sometimes included in the previous one (Barnett et al., 2017) and includes neighborhood resources and opportunities that serve people’s daily needs, particularly shops and local services (groceries, community pharmacies, recreation, health care, transportation, amenities, banks, post offices, administrative services, and social support). Finally, the social environment involves both the quality of relations in the neighborhood, measurable from the degree of trust, connectivity, and social cohesion perceived by residents (Cubbin et al., 2008), and social inequalities, measurable from the neighborhood’s income and socio-economic profile. While social theories largely support the idea that poor well-being may be linked to the community’s inability to develop common values and practices that regulate interpersonal relationships (Cantillon et al., 2003), with consequences for the social network and loneliness perception (Chen & Feeley, 2014; Pinquart & Sorensen, 2001), strong ties and solidarity between peers in deprived areas can also enhance individuals’ self-identity (Pinkster, 2007).

Some reviews, while focusing on older persons, have analyzed the impact of neighborhoods on physical health outcomes, demonstrating the role of exposure to green areas in reducing the risk of all-cause mortality and total cardiovascular disease (Yuan et al., 2020) or crime safety as a determinant of different health outcomes (Won et al., 2016). Other reviews have focused on negative states such as depression, highlighting the role of lack of green spaces, noise, and low quality of the built environment in depressive symptoms (Mair et al., 2008; Rautio et al., 2018) and underlining social dimensions, including socio-economic status, collective efficacy, and crime safety (Barnett et al., 2018). However, existing reviews have bypassed the aforementioned third approach to well-being—its flourishing and positive component—and focus only on one of the three environmental (physical, social, service) dimensions as explanatory factors. Finally, only quantitative studies have been examined in these reviews, neglecting older persons’ accounts of their perceptions and experiences, which are as essential as quantitative data to fully understand the environment/well-being relationship. The need to conduct a literature review to synthesize evidence in this area thus emerged. This study aims to fill this gap. Our objective is to detail existing knowledge on how urban neighborhood attributes influence the well-being of community-dwelling older persons.

## Methods

This review follows the Preferred Reporting Items for Systematic Reviews and Meta-Analyses (PRISMA) guidance for systematic reviews (Moher et al., 2009).

### Study Eligibility

Studies were considered eligible for the review if they: (i) were empirical studies assessing the effects of neighborhood attributes on older adults’ psychological well-being; (ii) defined neighborhood attributes based on subjectively or objectively measured features of the physical, social, and service environment as elaborated in the previous section; (iii) approached well-being based on subjective well-being, happiness, satisfaction with life, or the psychological component of quality of life; (iv) focused on community-dwelling older adults, defined as those aged 65+ (developed countries) or 60+ (in emerging countries) –we included other ages in cases where the studies identified the participants/sample as older people; (v) focused on urban or suburban areas (at least as part of the sample), controlling for the urban/rural location (quantitative studies) or providing sufficient information for distinguishing urban/rural settings (qualitative studies); (vi) were published in English, French, Spanish, Italian, or Portuguese. No restrictions were set regarding study design (quantitative, qualitative, and mixed-methods were all included) or publication year. Exclusion criteria were editorials, commentary and opinion pieces, literature reviews, and papers on theoretical issues. We also excluded studies focused on negative psychological conditions, such as depression or anxiety, as mentioned above.

### Search, Screening, and Data Extraction

Literature was sought using the following databases: ScienceDirect, Web of Science, Scopus, PubMed, EBSCO Academic Search Ultimate, and PsychINFO. We also manually searched issues published in the last 6 months from a selection of relevant journals from among the top 40 rankings of the 2016 SCImagoJR index of Geography and Planning, Transport, and Urban Studies publications. The following combination of search terms was used: [older people; older adults; older persons; seniors; elderly; elders; later life; aging; aging; old age] AND [built environment; neighborhood; outdoor environment; public space; community; amenities; local services] AND [wellbeing; well-being; happiness; subjective well-being; satisfaction with life; life satisfaction; quality of life]. The studies’ reference lists were reviewed to detect any studies that may have been missed. Relevant studies were then identified through two screening stages: (i) titles and abstracts and (ii) full texts. Studies were independently double screened by each team member, and differences were resolved through discussion and consensus. After the studies were selected, the data were extracted into a spreadsheet (Supplemental Appendix 1).

### Quality Appraisal

Two instruments were used for the quality assessment of the selected studies (detailed assessments are provided in Supplemental Appendix 2). Studies were assessed by the team members, and discrepancies were resolved through discussion. Quantitative studies were assessed using an eight-criteria instrument (Barnett et al., 2017; Cerin et al., 2017) (more details are available in Supplemental Appendix 2). Qualitative studies were assessed using the National Institute for Health and Care Excellence (NICE) checklist for external and internal validity and rated as ++, + or – (NICE, 2012). The NICE checklist has been widely used and is considered a reliable appraisal method (Baert et al., 2011; Kelly et al., 2016; Rushforth et al., 2016).

### Synthesis

Analysis was undertaken through an integrated approach, which is appropriate when qualitative and quantitative findings can be used to “confirm, extend, or refute each other” (Sandelowski et al., 2006). The findings are reported through a narrative synthesis. Narrative synthesis allows findings that include studies showing a high degree of heterogeneity in approaches, methods, outcomes, and explanatory factors to be summarized (Popay et al., 2006). Its advantages include the ability to reduce the complexity of disparate data into a more readable format (Popay et al., 2006). Meta-analysis was not possible owing to high heterogeneity in operational definitions and the measured outcomes. Quantitative studies were analyzed by grouping the variables associated with well-being outcomes into neighborhood dimensions (social, physical, and service attributes). For each variable identified, we assessed the number of studies that found significant, mixed, or no associations. Qualitative data were analyzed using a thematic analysis (Krippendorff, 2004). Findings from each qualitative study were copied verbatim into NVivo 12 (QSR International, 2018), a qualitative data analysis software program. A three-stage process was undertaken, based on line-by-line coding and grouping into previously defined categories and themes that were similar to the variables and dimensions of the quantitative studies. For example, the initial codes denoting “urban furniture” were grouped into a category called “walking and public space which was in turn integrated into the theme “physical environment. Finally, the results of the quantitative and qualitative syntheses were combined to yield a unified set of readable associations between variables.

## Results

### Overview of Included Studies

**Figure 1.**
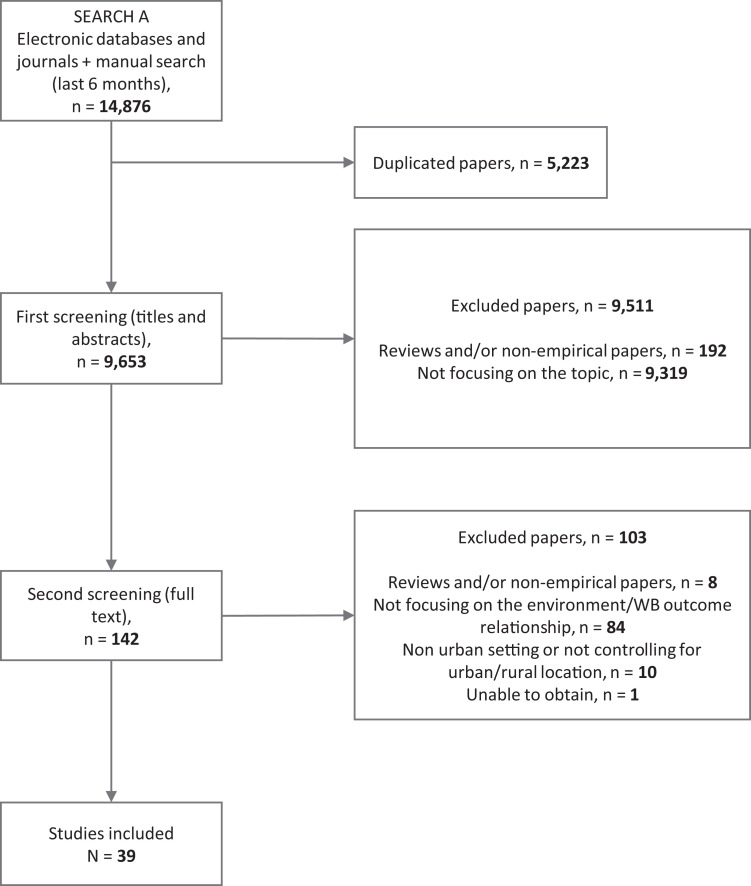
Prisma flow diagram.

A total of 14,876 references were gathered and assessed against the inclusion criteria. Ultimately, 39 studies were included in the review (Figure 1): 34 were quantitative studies (one represented by two different papers), five qualitative. The studies were predominantly published from 2011 onward (87%). China (n = 14), the UK (n = 6), and the US (n = 5) together account for 64% of all selected papers.

Quantitative studies included thirty cross-sectional studies, two longitudinal studies, and two studies implementing both designs (Table 1). Sample sizes varied from 36 to 9,965 persons (mean = 1,224, std = 1,982). Three studies included participants aged 50 or over, while the lower limit was 60 in 13 studies, 65 in six studies, and 70 in four studies. No study focused only on the oldest old group (85+). Among qualitative studies, sample sizes ranged from 11 to 121. Little information was provided regarding the participants’ age structure. One study mentioned the average age. The minimum age was 55 in two studies and 61, 65, and 69, respectively, with a maximum age exceeding 90 in three studies, reaching 89 in another study, and unknown in another study. Most participants were women, with percentages ranging from 55% to 81%. Participants living alone represented more than 50% in all qualitative studies. Two studies focused on low-income groups (Finlay et al., 2018) and two studies reported a considerable diversity of participants (Grant, 2007; Ottoni et al., 2016).

**Table 1.** Summary of Findings RELATED to variables.

Authors, year, country	Study Aims	Design	Sample, Setting	Main Findings	Quality Assessment
Au (2020, China)	To examine the psycho-social effects of the sense of community in mediating between WHO domains of age-friendliness and the life satisfaction of older adults.	Quantitative (Cross-sectional study)	898 people aged 55+, community centers, public area in housing estates, shopping areas, and parks	+ve: community support and health services, Brief Sense of Community Scale no effect: outdoor spaces and buildings, communication and information, transportation, place attachment	High
Barresi (1983, USA)	To assess the relationship between the residents’ evaluation of the environmental context and their well-being	Quantitative (Cross-sectional study)	2,262 people, age not stated, national sample	variable: perceived neighborhood safety, frequency of contact with neighbors, perceived neighborhood sociability	Moderate
Chang (2020, China)	To examine the relationship between physical environment and well-being from urban greenways in Taichung To examine how local older residents’ place attachment and perceived environmental stressors influence this relationship	Quantitative (Cross-sectional study)	769 people aged 55+, 13 greenways	+ve: place attachment (incl. social bonding, place identity, place dependence) −ve: environmental stressors (incl. trash, behaviour noise, traffic noise, air pollution)	High
Chapman (1983, USA)	To determine the extent to which environmental predictors explained the variance in five indicators of well-being: life satisfaction, activity level, social contacts, neighbor interaction, and neighborhood satisfaction	Quantitative (Cross-sectional study)	224 people aged 60+, elderly clients of local service agencies and living in target zones within the urban county	no effect: distance to services, age concentration (at both area and block levels), area social status, crime rate	Moderate
Cramm (2013, The Netherlands)	To investigate whether social capital and social cohesion within neighborhoods positively affect the well being of older adults	Quantitative (Cross-sectional study)	772 people aged 70+, urban neighborhoods in 4 districts	+ve: neighborhood-level social capital and social cohesion scores	High
Cramm (2014, The Netherlands)	To determine whether the neighborhood attributes solidarity and security positively affect the well-being of community-dwelling older people	Quantitative (Cross-sectional study)	869 people aged 70+, urban neighborhoods in four districts	+ve: solidarity within neighbourhood	Moderate
Cramm (2015, The Netherlands)	To identify longitudinal relationships between social cohesion and belonging and well-being	Quantitative (Longitudinal study)	945 (T0) and 588 (T1) people aged 70+, urban neighborhoods in 4 districts	+ve: social belonging and social cohesion scores	Moderate
Curl (2015, UK)	To examine how residents’ perceptions, behavior and wider quality of life outcomes have changed pre–post-DIY Street interventions by comparison with participants from non-intervention streets	Quantitative (Longitudinal study)	36 people aged 65+, 7 sites located in urban areas	no effect: knowing neighbors better or worse than two years previously	Moderate
Curl (2019, UK)	To examine: (i) how is the frequency of walking among older adults related to mental wellbeing; (ii) which social and built environment factors are important for older adults’ (a) wellbeing and (b) walking in deprived urban communities; (iii) whether the frequency of walking among older adults mediate between the social and built environment and wellbeing in a deprived urban context	Quantitative (Cross-sectional study)	1,071 people aged 60+, 15 deprived neighbourhoods	+ve: neighborhood problems (environment), neighborhood quality, feelings of safety in the dark −ve: neighborhood problems (incivilities) no effect: quality of local amenities	Moderate
Engel (2016, Canada)	To explore the association between the built environment and social cohesion with quality of life of low income, community-dwelling older adults	Quantitative (Cross-sectional study)	160 people aged 60+, 8 cities in Vancouver Metro Area	+ve: social cohesion scale −ve: street connectivity no effect: residential density, land-use mix, traffic hazards, aesthetics, walkability score, land-use (access), absence of cul-de-sacs, hilliness, physical barriers, crime	Low
Feng (2018, China)	To assess the relation between residential built environments and quality of life of older people	Quantitative (Cross-sectional study)	611 people aged 65+, 12 communities representing different built environments	+ve: satisfaction with residential environment, transport and social interaction no effect: built year, population density, informal space, walking environment, accessibility to local services	High
Gao (2017, China)	To examine the relationships of the social and physical attributes of a neighborhood with subjective well-being	Quantitative (Cross-sectional study)	2719 people aged 60+, 47 neighbourhoods	+ve: aesthetic quality score, social interaction with neighbors score (externally measured), social cohesion score (subjectively assessed) no effect: walkability score, social cohesion score (externally measured), social interaction with neighbors score (subjectively assessed)	Moderate
He (2020, China)	To examine the impact of satisfaction with transport systems on the social inclusion of older people and their wellbeing	Quantitative (Cross-sectional study)	271 people, age not stated (presumably 55+), One elderly center from each Hong Kong district	+ve: sense of community	Moderate
Lane (2020, Singapore)	To examine how neighborhood-based cognitive and structural social capital are associated with individual quality of life among a sample of community-dwelling older adults	Quantitative (Cross-sectional study)	981 people aged 55+, 9 residential neighbourhoods	+ve: neighborhood facilities, social cohesion	Moderate
Liu (2017, China)	To investigate the effects of residential environment and individual resources on the subjective well-being of older adults	Quantitative (Cross-sectional study)	1035 people aged 60+, one urban district, one rural county	+ve: access to health care and financial facilities −ve: access to entertainment, non-daily consumption facilities, proportion of older adults, educational and occupational status of neighborhood no effect: density of older adults	Moderate
Mottus (2012, UK)	To examine associations between neighborhood deprivation and various aspects of quality of life in older people	Quantitative (Cross-sectional study)	1091 people aged 68-71, city center and surroundings	−ve: deprivation	Moderate
Nieboer (2018, The Netherlands)	To identify relationships between age-friendly environments and older people’s overall well-being To identify the underlying instrumental goals to achieve overall well-being	Quantitative (Cross-sectional study)	588 people aged 70+, urban neighborhoods in 4 districts	+ve: housing, outdoor spaces and buildings, communication and information, community support and health services, transportation, respect and social approval, social participation variable: civic participation	High
Oswald (2011, Germany)	To investigate whether objective and perceived physical and social environmental aspects of the home and of the surrounding neighborhood represent resources for or risks to life satisfaction among young-old and old-old individuals	Quantitative (Cross-sectional study)	381 people aged 65+, one urban district	+ve: perceived outdoor place attachment	High
Paiva (2019, Portugal)	To study the relationship between the various domains of quality of life of the elderly and the eight aspects of age-friendliness	Quantitative (Cross-sectional study)	215 people aged 60+, one city center	+ve: community and health services, transportation no effect: outdoor spaces and buildings, information and communication	Moderate
Park (2017, South Korea)	To examine the role of environment on the well-being of vulnerable older adults	Quantitative (Cross-sectional study)	1655 people aged 65+, Seoul City	+ve: neighborhood overall evaluation, community and health services, transportation, social inclusion, social participation	Moderate
Smith (1995, Canada)	To examine whether psychological well-being of senior co-housing residents positively relate to their satisfaction with proximity to out of home service outlets, and whether those located in contrasting service environments exhibit different patterns of travel to service outlets	Quantitative (Cross-sectional study)	61 people aged 50+, 2 suburban sites with low rent senior citizen apartments projects	+ve: satisfaction with proximity to pharmacies, physicians’ offices, grocery stores, services no effect: satisfaction with proximity to bank / credit unions, and to public transit routes	Low
Sugiyama (2006, UK)	To assess whether a neighborhood environment facilitating older people’s outdoor activities has a positive effect on their well-being	Quantitative (Cross-sectional study)	58 people aged 65+, 4 cities (Edinburgh, Glasgow, Stockport and Cornwall)	+ve: natural and green environment scale, environmental support for outdoor activities	Low
Tiraphat (2017, Thailand)	To examine the association between age-friendly environments and quality of life among Thai older adults	Quantitative (Cross-sectional study)	4183 people aged 60+, national sample	+ve: social support, social cohesion, social trust, service accessibility, infrastructure and safety for walking and cycling, aesthetics (trash, litter) -ve: crime no effect: traffic hazards, street connectivity	High
Toma (2015, UK)	To examine whether perceived neighborhood factors were associated with positive well-being in older adults	Quantitative (Cross-sectional and longitudinal study)	6134 people aged 50+, national sample	−ve: neighbourhood disorder scale	Low
Ward Thompson (2014, UK)	To understand the influence of aspects of the built environment on older adults’ outdoor activity, wellbeing and quality of life To evaluate the effect of changes to the residential street environment on a cohort of older adults’ activity, well-being and quality of life To investigate the levels of physical activity in older adult participants in relation to self-reported outdoor activities	Quantitative (Pre-post cross-sectional study + longitudinal cohort study)	96 (T1), 61 (T2), 36 (T3), and 47 (activity survey) people aged 65+, 9 sites in urban areas in Scotland, England and Wales	+ve: good paths and cycleways, easy to get out and about, barriers/nuisance in local open space and neighborhood no effect: neighborhood tranquillity (more than 2 years before), bad footways/paths, pleasant local open space, knowing neighbors more than 2 years before	Low
Xie (2018, China)	To examine the extent to which older adults’ perceptions of environmental age-friendliness are associated with their life satisfaction	Quantitative (Cross-sectional study)	9965 people aged 60+, 1000 urban communities	+ve: local amenities, social inclusion no effect: community services	High
Yan (2014, China)	To examine the satisfaction of seniors in relation to the elderly services and living environments available to them	Quantitative (Cross-sectional study)	536 people aged 60+, six types of urban neighborhoods	+ve: accessibility to services	High
Yan (2015, China)	To identify which neighborhood factors concretely contribute to the life satisfaction of seniors	Quantitative (Cross-sectional study)	536 people aged 55+, representative communities in urban areas based on the authors’ own criteria/knowledge	+ve: accessibility to services	High
Yu (2019, China)	To explore the influence of outdoor living environment on the quality of life of elders living in old residential communities	Quantitative (Cross-sectional study)	107 people aged 60+, 6 urban districts	+ve: satisfaction with greenery, width, height no effect: satisfaction with accessibility, safety, road, seat	Moderate
Zhang (2019, China)	To examine the associations between objectively-measured neighborhood physical environmental attributes and quality of life domains in Hong Kong older community dwellers and estimate the moderating effects of neighborhood environmental attributes on the associations of living arrangements with quality of life	Quantitative (Cross-sectional study)	909 people aged 60+, Hong Kong neighborhoods	+ve: entertainment density (curvilinear) −ve: litter/decay, street intersection density variable: signs of crime / disorder (depending upon living arrangement) no effect: residential density, traffic safety, pollution, number of parks, park area, activity types in the park, trees in park, paths in park, park aesthetics, park visibility, greenery/natural sights, connectivity, pedestrian infrastructure, sitting facilities, health clinics/services, civic and institutional density, prevalence of non-food retail / services, recreation density, prevalence of food-related shops, eating outlets, public transport stops, area-level SES	High
Zhang (2019, China)	To explore how the urban neighborhood environment affects quality of life of community-dwelling older adults and develop a mediation model called “Neighborhood Environment-Quality of Life (NE-QoL)” for community-dwelling older adults	Quantitative (Cross-sectional study)	192 people aged 60+, Nanjing neighborhoods	+ve: natural environment, sidewalk condition, facilities related to daily life, neighbor support no effect: land-use mix, barrier-free design, street condition, outdoor public spaces, traffic-related safety, design-related safety, facilities related to physical exercise and recreation, accessibility to facilities, public transport, crime-related safety	Moderate
Zhang (2020, USA)	To examine the associations of neighborhood cohesion with psychological distress and life satisfaction as well as the mediating role of resilience and the moderating role of birth place in the associations	Quantitative (Cross-sectional study)	430 people aged 55+, urban neighbourhoods	+ve: neighborhood social cohesion	Moderate
Zhang (2017, China)	To examine the relationship between a sense of community and life satisfaction as moderated by personal resilience and partner resilience among older adults	Quantitative (Cross-sectional study)	516 (258 couples) people aged 60+, 22 communities	+ve: sense of community	Moderate
Zhang (2017, China)	To investigate the relationship between perceived neighborhood environment and subjective well-being and the mediating effect of a sense of community among elderly	Quantitative (Cross-sectional study)	720 people aged 50+, 11 urban neighborhoods in the 3 cities	+ve: sense of community	High
Coleman (2015, New Zealand)	To examine how bluespaces might contribute to experiences of healing and wellbeing	Qualitative (Phenomenological interpretive perspective)	28 people aged 65-94, "suburban" island near metro area	+ve: natural areas, health care services, local services, transit, sense of community	High
Grant (2007, New Zealand)	To examine how the day-to-day experiences of elderly people living in retirement villages influence their lifestyle and quality of life	Qualitative (Phenomenology)	121 people aged 69-91, 12 retirement villages	+ve: local services, age homogeneity, sense of community	High
Keene (2013, USA)	To discuss the sense of belonging and kinship that some older adults attribute to living in public housing communities that were “like families” and where they often held important roles as respected elders To discuss the challenges that they contend with as these networks are scattered through demolition and relocation	Qualitative (Modified grounded theory approach)	25 people aged 55+, 7 public housing communities (recently demolished family and senior developments, one ‘control’ not demolished senior development)	+ve: local services, transit −ve: age homogeneity variable: deprivation	High
Ottoni (2016, Canada)	To examine how one microscale feature (benches) influence older adults experiences of mobility and well-being To explore how these experiences affect and are affected by the social environment of the neighborhoods where older adults live	Qualitative (Phenomenology)	28 (T1) and 22 (T2) people aged 61-89, 3 adjacent urban neighborhoods: Vancouver’s West End, Yaletown and Downtown	+ve: natural areas, walking and public space, local services, sense of community	High
Finlay (2018, USA)	To characterize salient features of built and social environments that are essential to support low-income ageing residents	Qualitative (Thematic analysis)	38 people aged 55-92, 3 socio-economic and geographic case studies of the Minneapolis metropolitan area	+ve: walking and public space, health care services, local services, ethnic diversity −ve: density, age homogeneity variable: deprivation	High

In quantitative studies, outcome measures were addressed in several ways. Twelve studies analyzed well-being based on various instruments (ad hoc instruments, ICECAP-O, MIL, PERMA model, PWI, SPF-IL, and WEMBS; see Supplemental Appendix 3 for the full list of instruments and explanation of acronyms). Fourteen studies analyzed life satisfaction based on LSI-A or B, SWLS, or ad hoc instruments. Finally, ten studies analyzed quality of life using the WHOQOL, the WHOQOL-Bref, or the CASP-12 or 19.

Twelve domains of independent variables emerged from the quantitative studies: alongside sociodemographic variables (included in all studies), social environmental attributes were the most frequently incorporated (32 studies), followed by physical (26 studies) and service (25) environment attributes. Twenty studies reported on all three dimensions of the environment. In all three of the social, physical, and service environmental domains, subjective evaluations were preferred (29, 24, and 21 studies, respectively) over more objective items (four, six, and four studies, respectively). Only one study simultaneously examined objective and subjective variables related to social environmental attributes (Gao et al., 2017) whereas four did so based on physical attributes (Chang et al., 2020; Engel et al., 2016; Feng et al., 2018; Gao et al., 2017). No study examined both variable types in the context of a service environment. All qualitative studies included elements of the three dimensions.

### Quality of the Studies

All qualitative studies were deemed to be of high quality. Twelve quantitative studies were considered to be of high quality and 17 were deemed to be of moderate quality. Lower quality levels among the quantitative studies were related to low participant response rate, fewer sociodemographic covariates and housing-related variables, and failure to incorporate at least one major dimension (15 studies did not include all social, physical, and service environments).

### Synthesis of the Findings

#### Physical environment: Access to natural areas

The most salient feature in the studies was the presence of and access to natural areas, such as green and blue spaces (Table 2—a more detailed table is provided in Supplemental Appendix 4). Natural areas were associated with well-being in three out of four quantitative studies (Sugiyama & Thompson, 2006; Yu et al., 2019; Zhang & Li, 2019a, 2019b). These spaces were also strongly associated with enjoyment and reflection in qualitative studies, as they provide pleasurable passive interactions, including observing people, engaging in small talk, or sometimes offering small snacks (e.g., fruit) to others (Coleman & Kearns, 2015; Ottoni et al., 2016). Natural areas may also have psychological healing functions. For example, one 83-year-old participant mentioned that the daily presence of the sea helped her to better accept her own physical decline, while an 81-year-old woman indicated that it helped her to overcome past experiences and recent losses (Coleman & Kearns, 2015).

**Table 2.** Summary of Findings Related to Variables.

**Environmental Domain**	**Category of the Variables**	**Number of Studies**	**% of Studies**	**Positive Effect**	**No Effect Found**	**Variable or Ambiguous Effect(s)**	**Negative Effect**
Physical	Cleanliness	3	8%	3	0	0	0
	Density and urban fabric	9	23%	1	6	0	2
	Natural areas	6	15%	5	1	0	0
	Overall evaluation	4	10%	3	1	0	0
	Overall neighborhood environment	1	3%	1	0	0	0
	Walk. and public space	15	38%	5	4	6	0
Service	Health care	7	15%	6	1	0	0
	Local services	15	21%	8	3	4	0
	Social support	5	5%	2	3	0	0
	Transit	13	23%	9	4	0	0
Social	Age homogeneity	5	13%	1	1	0	3
	Deprivation	6	15%	0	2	2	2
	Ethnic diversity	1	3%	1	0	0	0
	Overall evaluation	2	5%	1	1	0	0
	Security	8	21%	3	3	2	0
	Sense of community	24	62%	19	2	3	0

The association of well-being with quality of space for walking and public interaction was variable, with three quantitative studies finding positive effects (Curl & Mason, 2019; Nieboer & Cramm, 2018; Sugiyama & Thompson, 2006), six finding variable effects, and four finding no association. Variability was caused by the variables used: studies with many items describing walking and public spaces found more variable results, while more global indicators were generally positively associated with well-being. The absence of barriers, good paths, and traffic segregation were identified as positive (Ward Thompson et al., 2014) along with pavement quality (Finlay et al., 2018), safe intersections and pedestrian signals allowing sufficient time to cross the street (Finlay et al., 2018), and benches installed at regular distances, offering the possibility of rest (Ottoni et al., 2016). This is because they support daily activities, such as shopping, and enhance social interactions and conviviality or quietude (Coleman & Kearns, 2015; Ottoni et al., 2016). When included as a variable, cleanliness was also highlighted as positively associated with well-being (Curl & Mason, 2019; Tiraphat et al., 2017; Zhang et al., 2019).

Seasonal variability may compromise older adults’ mobility (Finlay et al., 2018; Ottoni et al., 2016). In particular, intense heat, wind, snow, and icy conditions in winter were strong deterrents that exacerbated feelings of vulnerability (Finlay et al., 2018).

#### Social environment: Sense of community

The social environment was mainly assessed through four groups of variables: area-level deprivation, age homogeneity, perception of security, and sense of community.

Sense of community was overwhelmingly found to have a positive effect in 14 out of 19 quantitative studies (Au et al., 2020; Chang et al., 2020; Cramm & Nieboer, 2014, 2015; Cramm et al., 2013; Engel et al., 2016; Feng et al., 2018; He et al., 2020; Park & Lee, 2017; Tiraphat et al., 2017; Xie, 2018; Zhang & Li, 2019a, 2019b; Zhang et al., 2020; Zhang & Zhang, 2017), while three studies found variable effects and two found no effect. Variability in the findings was related to the measuring instrument, as self-reported appraisals yielded more positive results than external assessments. Qualitative studies strongly highlighted the importance of the sense of community, as social connectedness is fed by reciprocal relationships and mutual assistance. In some cases, older participants emphasized the possibility of getting help, and several participants viewed their neighborhood as a family.

Neighborhoods’ deprivation levels were negatively associated with well-being in only one study (Liu et al., 2017), while two studies found no association (Chapman & Beaudet, 1983; Zhang et al., 2019). In qualitative studies, such neighborhoods were, in some cases, associated with stronger local ties and support, which may explain the relative lack of negative associations. A sense of social class solidarity may emerge in some cases with strong ties and peer support (Finlay et al., 2018; Keene & Ruel, 2013). In some cases, relocation of older adults from public housing neighborhoods to high-profile residential settings was experienced as a setback, as gains in residential comfort did not compensate for losses in social ties. This was exemplified by a 77-year-old woman who was “desperately unhappy and preferred the old entirely subsidized model because of the organized social activities and sense of community” (Finlay et al., 2018, p. 17).

Two quantitative studies included age homogeneity as a variable. One found no significant relationships with well-being (Chapman & Beaudet, 1983), while the other observed a negative effect (Liu et al., 2017). However, one qualitative study on retirement communities stated that peer support may be regarded as a means of maintaining or restoring a sense of social identity and avoiding ageist attitudes experienced elsewhere (Grant, 2007). This contrasts with two studies that suggested the importance of multigenerational networks in the vicinity, as they can contribute to maintaining the sense of performing a social role (Finlay et al., 2018; Keene & Ruel, 2013). Local ties may be associated with multigenerational networks in neighborhoods where older adults receive help from younger people and/or provide support to them, for example, by looking after children (Keene & Ruel, 2013). In other cases, kinship outweighed dwelling conditions in self-perceptions and nurtured a sense of common belonging shared with people living through the same experience of age and declining health (Finlay et al., 2018).

Finally, security was positively associated with well-being in two studies (Curl & Mason, 2019; Tiraphat et al., 2017), with three studies finding no effect. In two studies, the relationship was moderated by other variables: security was associated with well-being only among women in one study (Barresi et al., 1983), and among persons living alone in another one (Zhang et al., 2019).

#### Service environment: Transit services

Transit services were associated with well-being in six out of 10 studies (Feng et al., 2018; Nieboer & Cramm, 2018; Paiva et al., 2019; Park & Lee, 2017; B. Yan et al., 2015; Yan et al., 2014). In the remaining four, no significant effect was identified (Au et al., 2020; Smith & Gauthier, 1995; Zhang et al., 2019; Zhang & Li, 2019a, 2019b). The importance of good transit services and low floor vehicles was also highlighted in two qualitative studies (Coleman & Kearns, 2015; Finlay et al., 2018; Keene & Ruel, 2013).

Social support, as a service provided by institutions, was associated with well-being in two out of five studies (Nieboer & Cramm, 2018; Park & Lee, 2017), while three studies observed no relationship (Au et al., 2020; Paiva et al., 2019; Xie, 2018). Healthcare access was more salient, as four studies out of five found a positive relationship with well-being (Au et al., 2020; Liu et al., 2017; Paiva et al., 2019; Smith & Gauthier, 1995). Participants in one qualitative study even indicated that reduced access to care could cause relocation in case of declining health (Keene & Ruel, 2013).

Finally, local services, including food retailers and coffee shops, were associated with well-being in only three studies (Lane et al., 2020; Tiraphat et al., 2017; Xie, 2018), while three studies found no effect and four studies found variable effects due to the inclusion of several service types in the variables. However, local services were frequently mentioned in qualitative studies as they provide a sense of choice and freedom and, thus, the possibility for people to “escape the stereotypes of old age” (Grant, 2007) as a means of more easily accessing help when needed and wanted, and facilitate meaningful social interactions or small talk (Finlay et al., 2018; Ottoni et al., 2016). A 74-year-old participant offered a good example of the social function of local sites such as coffee shops, as they “represented a site of comfort, care, and attention, and one of the primary reasons she enjoyed her apartment and wanted to remain living there” (Finlay et al., 2018, p. 14).

#### Individual variables: The sense of belonging

Half of the studies suggested that familiarity and a sense of history, normally resulting from a long-standing relationship with the place of residence, helped bolster a sense of belonging and attachment to a place (Elliott et al., 2014; Finlay et al., 2018; Keene & Ruel, 2013). Older adults could rely on their deep knowledge of their social and physical environment to compensate for hazardous living conditions and associated health risks (Finlay et al., 2018) and to reduce their perception of crime and insecurity (Finlay et al., 2018). Long-term kinship was substituted by new residents with less local rooting and higher levels of car-based mobility (Elliott et al., 2014; Finlay et al., 2018). This is why residential relocation was associated with a painful loss of daily and meaningful relationships, a situation that can be overcome by speaking with friends/relatives by telephone (Keene & Ruel, 2013) or by maintaining habits, such as attending the same church (Keene & Ruel, 2013) as before. New contacts and support were difficult to establish in the new neighborhood (Finlay et al., 2018) unless the new place was age-segregated, such as a senior high-rise (Finlay et al., 2018; Keene & Ruel, 2013) or retirement community (Grant, 2007). Poor transport as well as age and health limitations accentuated the feeling of loss.

The studies demonstrated that as individuals progressed in the aging process, health deterioration led to exclusionary processes and a loss of autonomy and perceived competence (Coleman & Kearns, 2015; Keene & Ruel, 2013). It could also lead to forced relocation and disengagement from decision making (Coleman & Kearns, 2015). In addition, the loss of a spouse could lead to isolation and grief (Coleman & Kearns, 2015) and even safety/security fears (Finlay et al., 2018). Age also appeared to be associated with a greater sense of attachment to place (Finlay et al., 2018) and attention to the landscape (Coleman & Kearns, 2015).

## Discussion

This systematic review sought to examine the relationship between neighborhood characteristics and the well-being of older adults living in urban areas. We identified 39 studies (34 quantitative, five qualitative), of which 56% were published from 2016 onward. Our findings indicate that some features are consistently (although not entirely) associated with older adults’ well-being, including the presence and availability of natural areas and of adequate street furniture (physical environment); the sense of community (social environment), which may compensate for adverse living conditions and area-level social deprivation; and good transit and the availability of local services (service environment).

Our findings can therefore contribute to improved planning practice and decisions with potential impacts on the quality of aging in urban areas. Our findings highlight the necessity of increasing high-quality green (and/or blue) areas as well as street furniture (including benches and tables) that may support older adults’ daily activities. Trees and other weather-related structures should be strategically and regularly placed in public spaces. Benches should be more ubiquitous in streets and better distributed along adequately maintained sidewalks, as they also fulfill utilitarian purposes (as intermediate rest spots when going shopping, for example). Social activities entailing the active or passive participation of older adults should also be promoted.

This review has also highlighted several theoretical and methodological limitations to how environmental variables are considered. First, the simultaneous incorporation of the three dimensions (physical, social, services) is not always achieved. It is therefore possible that the interactions between the various environmental variables have not been fully explored. For example, the sense of community can play a countervailing role in neighborhoods with poor service provision. In other cases, street furniture can reinforce perceptions of safety and sense of community as well as supporting service use (Mehta, 2009). Second, these same indicators may be based on a subjective assessment of the older persons or on external evaluation conducted by the researcher or the official data-producing institution (we avoid the term “objective” insofar as even an external measure can be based on a non-objective assessment). This difference is important because of the possible bias and interactions between environmental variables and the respondent’s position. However, few studies have taken these differences into account. Third, the studies reveal a lack of consistency regarding how neighborhood attributes are defined, which at times has challenged the categorization of environmental variables: for example, several studies included personal or housing-related items in the environmental assessment (Curl et al., 2015; J. Yan et al., 2014, 2015; Lane et al., 2020).

Adjustments for individual characteristics also varied across studies. While all studies included sex, age, and socio-economic variables, not all included adjustments for housing condition, functional status, social capital, family functioning, psychological traits, physical activity, or adverse life events. These variables can play a significant role in moderating the relationship between the environment and well-being.

This review’s findings highlight several avenues for future research. Some are methodological and are directly linked to the above. The incorporation of variables representing the three physical, social, and service dimensions and ensuring that subjectively assessed factors do not introduce bias are paramount. Additionally, more longitudinal designs are needed to better understand the causal relationships between well-being and the environment (Renalds et al., 2010; Rosso et al., 2011; Yen et al., 2009). As 88% of quantitative studies were cross-sectional, it is difficult at this stage to exclude the role of residential self-selection in the well-being outcomes (e.g., an older adult with high income and high level of well-being choosing to live in a neighborhood with good conditions) (Barnett et al., 2017; Cerin et al., 2017). Considering their slight presence in this review, more qualitative studies are also needed to further explore the role of the different environmental features identified in this review. Alongside these methodological considerations, future research should acknowledge the diversity of the older population and pay greater attention to specific age groups (e.g., the so-called “young old,” “middle old,” and “old old” groups), to social groups (low-income, minorities), and to people with physical and/or cognitive impairments. It is clear, based on this review, that these distinctions remain rare in the current literature.

This review has several limitations. As in most systematic reviews, selection bias may have arisen from the restrictions imposed on the search. A second limitation relates to an inevitable level of subjectivity in the exercise of quality appraisal. Precautions were taken to minimize the risk of subjective evaluation, mainly through the description of the ratings and methods used to evaluate the studies’ quality. Finally, we were unable to accurately quantify associations between variables and well-being-related outcomes owing to the significant variability in covariates and measured outcomes.

## Conclusion

Environments are instrumental in supporting older adults’ feelings of comfort and competence through the existence and availability of a wide range of services and activities, physical support for daily mobility and routines, and a good social network. This review’s findings reveal some of the main environmental factors that support older adults’ well-being in urban areas and thus provide some insights that may benefit planning practice and decision-makers. They also show that the exclusion of some dimensions from the analysis may obscure the complexity of the interactions between older adults and their environment. Further research is thus necessary to explore this complexity more fully and to better guide future decisions with increasing impacts in an aging population.

## Supplemental Material

Supplemental Material, sj-pdf-1-roa-10.1177_0164027521999980 - Neighborhood Attributes and Well-Being Among Older Adults in Urban Areas: A Mixed-Methods Systematic Review Click here for additional data file. Supplemental Material, sj-pdf-1-roa-10.1177_0164027521999980 for Neighborhood Attributes and Well-Being Among Older Adults in Urban Areas: A Mixed-Methods Systematic Review by Miguel Padeiro, José de São José, Carla Amado, Liliana Sousa, Carla Roma Oliveira, Alina Esteves and Jennifer McGarrigle in Research on Aging

Supplemental Material, sj-pdf-2-roa-10.1177_0164027521999980 - Neighborhood Attributes and Well-Being Among Older Adults in Urban Areas: A Mixed-Methods Systematic Review Click here for additional data file. Supplemental Material, sj-pdf-2-roa-10.1177_0164027521999980 for Neighborhood Attributes and Well-Being Among Older Adults in Urban Areas: A Mixed-Methods Systematic Review by Miguel Padeiro, José de São José, Carla Amado, Liliana Sousa, Carla Roma Oliveira, Alina Esteves and Jennifer McGarrigle in Research on Aging

Supplemental Material, sj-pdf-3-roa-10.1177_0164027521999980 - Neighborhood Attributes and Well-Being Among Older Adults in Urban Areas: A Mixed-Methods Systematic Review Click here for additional data file. Supplemental Material, sj-pdf-3-roa-10.1177_0164027521999980 for Neighborhood Attributes and Well-Being Among Older Adults in Urban Areas: A Mixed-Methods Systematic Review by Miguel Padeiro, José de São José, Carla Amado, Liliana Sousa, Carla Roma Oliveira, Alina Esteves and Jennifer McGarrigle in Research on Aging

Supplemental Material, sj-pdf-4-roa-10.1177_0164027521999980 - Neighborhood Attributes and Well-Being Among Older Adults in Urban Areas: A Mixed-Methods Systematic Review Click here for additional data file. Supplemental Material, sj-pdf-4-roa-10.1177_0164027521999980 for Neighborhood Attributes and Well-Being Among Older Adults in Urban Areas: A Mixed-Methods Systematic Review by Miguel Padeiro, José de São José, Carla Amado, Liliana Sousa, Carla Roma Oliveira, Alina Esteves and Jennifer McGarrigle in Research on Aging
