# Supplementary material for: Neighborhood Attributes and Well-Being Among Older Adults in Urban Areas: A Mixed-Methods Systematic Review
Source: Res Aging. 2021 Apr 28;44(5-6):351–68. doi: 10.1177/0164027521999980 (PMC9039320; doi:10.1177/0164027521999980)
Supplement: Supplemental Material, sj-pdf-1-roa-10.1177_0164027521999980 - Neighborhood Attributes and Well-Being Among Older Adults in Urban Areas: A Mixed-Methods Systematic Review [file sj-pdf-1-roa-10.1177_0164027521999980.pdf]

Data extraction items

**Category**	**Items**
Publication details	Record code<br>First Author<br>Year<br>Type of publication<br>Title of the paper
Aims/objectives	Aims/objectives
Theoretical/Conceptual framework	Conceptual definition of well-being<br>Operationalisation of the concept of well-being<br>Definitions of other relevant concepts<br>Theoretical framework (if any)
Research methods	Method<br>Study design<br>Location<br>Type of settings<br>Does the study focus on a recent intervention?<br>Details on the recent intervention<br>Sampling technique (study sites)<br>Sample composition for settings<br>Sampling technique for participants<br>Sample dimension (participants)<br>More details on the characteristics defining the participants (age, gender, health condition, ethnicity, etc.)<br>Data collection technique<br>Data analysis technique(s)<br>Outcome(s) measured<br>Physical outdoor environment attributes included as descriptors and/or independent variables (does not include home)<br>Social environment attributes included as descriptors and/or independent variables<br>Service environment attributes included as descriptors and/or independent variables<br>Other co-variates (including home-related ones)<br>Length of follow-up, number/times of follow up (if necessary)
Findings	Main findings<br>More details on the relationship between physical environment and subjective well-being, if needed<br>More details on the relationship between social environment and subjective well-being, if needed<br>More details on the relationship between service environment and subjective well-being, if needed<br>Is there another relevant variable to be mentioned? If yes, which one and how is it relevant?<br>More details of outcomes and findings, if any
Observations	Observations
