# Supplementary material for: Neighborhood Attributes and Well-Being Among Older Adults in Urban Areas: A Mixed-Methods Systematic Review
Source: Res Aging. 2021 Apr 28;44(5-6):351–68. doi: 10.1177/0164027521999980 (PMC9039320; doi:10.1177/0164027521999980)
Supplement: Supplemental Material, sj-pdf-2-roa-10.1177_0164027521999980 - Neighborhood Attributes and Well-Being Among Older Adults in Urban Areas: A Mixed-Methods Systematic Review [file sj-pdf-2-roa-10.1177_0164027521999980.pdf]

Detailed assessment

Quantitative studies

Study	Design	Participant response rate	Number of participants	Validity and reliability of outcome measures	Adjustment for key socio-demographic covariates (at least age, sex, marital status, SES and education considered)	Adjustment for other covariates (at least housing-related covariates considered)	All 3 dimensions of environment included	Robustness and sensitivity tests described	TOTAL	Quality assessment
Au (2020, China)	Cross-sectional	0	1	1	1	1	1	0.5	5.5	High
Barresi (1983, USA)	Cross-sectional	1	1	1	0.5	1	0	0	4.5	Moderate
Chang (2020, China)	Cross-sectional	1	1	1	0.5	0.5	1	1	6	High
Chapman (1983, USA)	Cross-sectional	0	0.5	1	0.5	1	1	0	4	Moderate
Cramm (2013, The Netherlands)	Cross-sectional	0.5	1	1	1	1	1	1	6.5	High
Cramm (2014, The Netherlands)	Cross-sectional	0	1	1	1	0	0	1	4	Moderate
Cramm (2015, The Netherlands)	Longitudinal	0	1	1	0.5	0	0	1	3.5	Moderate
Curl (2015, UK)	Longitudinal	0	0	1	0	1	1	1	4	Moderate
Curl (2019, UK)	Cross-sectional	0	1	1	1	0	1	1	5	Moderate
Engel (2016, Canada)	Cross-sectional	0	0	1	0	0	0	1	2	Low
Feng (2018, China)	Cross-sectional	1	1	1	0.5	0	1	1	5.5	High
Gao (2017, China)	Cross-sectional	1	1	1	0.5	0	0	1	4.5	Moderate
He (2020, China)	Cross-sectional	0	0.5	1	0.5	0.5	1	1	4.5	Moderate
Lane (2020, Singapore)	Cross-sectional	0	1	1	1	1	0.5	0.5	5	Moderate
Liu (2017, China)	Cross-sectional	0	1	1	0.5	1	0	1	4.5	Moderate
Mottus (2012, UK)	Cross-sectional	1	1	1	0	0	1	1	5	Moderate
Nieboer (2018, The Netherlands)	Cross-sectional	1	1	1	1	0	1	1	6	High
Oswald (2011, Germany)	Cross-sectional	1	0.5	1	0	1	1	1	5.5	High
Paiva (2019, Portugal)	Cross-sectional	0	0.5	1	0	0.5	1	1	4	Moderate
Park (2017, South Korea)	Cross-sectional	1	1	0	0.5	1	1	0	4.5	Moderate
Smith (1995, Canada)	Cross-sectional	0	0	1	1	0	0	0	2	Low
Sugiyama (2006, UK)	Cross-sectional	1	0	1	0	0	0	1	3	Low
Tiraphat (2017, Thailand)	Cross-sectional	1	1	1	1	0.5	1	0	5.5	High
Toma (2015, UK)	Longitudinal	0	1	1	1	0	0	0	3	Low
Ward Thompson (2014, UK)	Longitudinal	0	0	1	1	0	0	0	2	Low
Xie (2018, China)	Cross-sectional	1	1	1	0.5	1	0	1	5.5	High
Yan (2014, China)	Cross-sectional	1	1	1	1	1	1	1	7	High
Yan (2015, China)	Cross-sectional	1	1	1	0	1	1	1	6	High
Yu (2019, China)	Cross-sectional	1	0	1	0.5	0	1	0.5	4	Moderate
Zhang (2019, China)	Cross-sectional	0.5	1	1	0.5	1	1	0.5	5.5	High
Zhang (2019, China)	Cross-sectional	1	0	1	0	0.5	1	1	4.5	Moderate

Zhang (2020, USA)	Cross-sectional	0	0.5	1	1	0.5	0	0.5	3.5	Moderate
Zhang (2017, China)	Cross-sectional	1	1	1	1	0	0	0	4	Moderate
Zhang (2017, China)	Cross-sectional	1	1	1	1	0	1	1	6	High
	**TOTAL**	**17**	**22**	**33**	**14**	**13**	**20**	**21**		
	**TOTAL %**	**50%**	**65%**	**97%**	**41%**	**38%**	**59%**	**62%**		

Quality score calculation

(1) participant response rate or evidence of a representative sample [≥ 80% = 1, 60–80% = 0.5, <60% = 0]; (2) number of participants included in the analysis [≥ 500 = 1, 200–500 = 0.5, <200 = 0]; (3) validity and reliability of outcome measures based on the described metric properties of the instrument used or outcome measures well-established in the field [yes = 1, partially = 0.5, no/unclear = 0]; (4) adjustment for key socio-demographic covariates (at least age, sex, marital status, and education) [yes = 1, partially (50–80%) = 0.5, no/unclear = 0]; (5) adjustment for other covariates (at least housing-related covariates) [yes = 1, partially (50–80%) = 0.5, no/unclear = 0]; (6) adjustment for covariates from all three environmental dimensions (social, physical, and services) [yes = 1, partially (2 dimensions) = 0.5, no/unclear = 0]; and (7) robustness and sensitivity tests are described [yes = 1, partially (50–80%) = 0.5, no/unclear = 0]. Criteria do not include distinction between cross-sectional, longitudinal and quasi-experimental design, because almost all studies were cross-sectional. Scores on the above items were summed: studies with a score ranging from 0 to 3.49 were deemed low quality, scores from 3.5 to 5.49 were deemed moderate quality, and scores equal to 5.5 or over were deemed high quality.

Qualitative studies

**Publication details**	**Theoretical approach**		**Study design**	**Data collection**	**Trustworthiness**			**Analysis**						**Ethics**	**TOTAL**	**Quality assessment**
Record code	Is a qualitative approach appropriate?	Is the study clear in what it seeks to do?	How defensible/rigorous is the research design/methodology?	How well was the data collection carried out?	Is the role of the researcher clearly described?	Is the context clearly described?	Were the methods reliable?	Is the data analysis sufficiently rigorous?	Is the data 'rich'?	Is the analysis reliable?	Are the findings convincing?	Are the findings relevant to the aims of the study?	Conclusions	How clear and coherent is the reporting of ethics?		
COL15	1	1	1	1	0	1	1	1	1	1	1	1	1	0,5	**12,5**	High
FIN18	1	0,5	1	1	0	1	1	1	1	1	1	1	1	0,5	**12**	High
GRA07	1	1	1	1	1	1	0	1	1	1	1	1	1	1	**13**	High
KEE13	1	1	1	1	0,5	1	0,5	1	1	0	1	1	0,5	0	**10,5**	High
OTT16	1	1	1	1	1	1	1	1	1	1	1	1	1	1	**14**	High
