# Supplementary material for: Neighborhood Attributes and Well-Being Among Older Adults in Urban Areas: A Mixed-Methods Systematic Review
Source: Res Aging. 2021 Apr 28;44(5-6):351–68. doi: 10.1177/0164027521999980 (PMC9039320; doi:10.1177/0164027521999980)
Supplement: Supplemental Material, sj-pdf-3-roa-10.1177_0164027521999980 - Neighborhood Attributes and Well-Being Among Older Adults in Urban Areas: A Mixed-Methods Systematic Review [file sj-pdf-3-roa-10.1177_0164027521999980.pdf]

Outcomes and instruments in quantitative studies

Studies	Outcomes addressed	Instruments	Description
Au (2020, China)	Life satisfaction	SWLS	5-item Satisfaction with Life Scale
Barresi (1983, USA)	Well-being	Ad hoc instrument	Combination of two single-item based dimensions: general life satisfaction and happiness
Chang (2020, China)	Well-being	PERMA model	15-item questionnaire based on Seligman’s (2011) PERMA model, comprising five aspects of well-being: positive emotion; engagement; positive relationships; meaning; and accomplishment
Chapman (1983, USA)	Life satisfaction	LSI-A	20-item Life Satisfaction Index-A
Cramm (2013, The Netherlands)	Well-being	SPF-IL	15-item version of the Social Production Function Instrument for the Level of Well-being
Cramm (2014, The Netherlands)	Well-being	SPF-IL	15-item version of the Social Production Function Instrument for the Level of Well-being
Cramm (2015, The Netherlands)	Well-being	SPF-IL	15-item version of the Social Production Function Instrument for the Level of Well-being
Curl (2015, UK)	Quality of life	CASP-19	19-item Control, Autonomy, Self-realization, and Pleasure Scale
Curl (2019, UK)	Well-being	WEMBS	Warwick–Edinburgh Mental Wellbeing Scale
Engel (2016, Canada)	Well-being	ICECAP-O	Capability well-being
Feng (2018, China)	Life satisfaction	SWLS	5-item Satisfaction with Life Scale
Gao (2017, China)	Well-being	PWI	Chinese version of the Personal Wellbeing Index
He (2020, China)	Well-being	Ad hoc instrument	8-item instrument
Lane (2020, Singapore)	Quality of life	CASP-12	12-item Control, Autonomy, Self-realization, and Pleasure Scale
Liu (2017, China)	Well-being	Ad hoc instrument	Subjective well-being score
Mottus (2012, UK)	Quality of Life	WHOQOL-BREF	World Health Organization Quality of Life Instruments - Bref
Nieboer (2018, The Netherlands)	Well-being	SPF-IL	15-item version of the Social Production Function Instrument for the Level of Well-being
Oswald (2011, Germany)	Life satisfaction	SWLS	Cognitive dimension of the Satisfaction with Life Scale
Paiva (2019, Portugal)	Quality of life	WHOQOL-BREF	World Health Organization Quality of Life Instruments - Bref
Park (2017, South Korea)	Life satisfaction	Ad hoc instrument	Life satisfaction index based on satisfaction with: (i) public transportation system; (ii) housing conditions; (iii) family relationships; (iv) relationships with friends and relatives; (v) cultural activities; (vi) social participation; (vii) economic status; (viii) personal health; (ix) information and services provided by public agencies in the community.
Smith (1995, Canada)	Life satisfaction	LSI-B	13-item Life Satisfaction Index B (LSI-B)
Sugiyama (2006, UK)	Life satisfaction	SWLS	5-item Satisfaction with Life Scale (SWLS)
Tiraphat (2017, Thailand)	Quality of life	WHOQOL-BREF	World Health Organization Quality of Life Instruments - Bref
Toma (2015, UK)	Life satisfaction	SWLS	5-item Satisfaction with Life Scale
Toma (2015, UK)	Quality of life	CASP-19	19-item Control, Autonomy, Self-realization, and Pleasure Scale

Ward Thompson (2014, UK)	Quality of life	CASP-19	19-item Control, Autonomy, Self-realization, and Pleasure Scale
Xie (2018, China)	Life satisfaction	Single item	Single item: “Overall, how satisfied are you with your present life?”
Yan (2014, China)	Life satisfaction	Single item	Single item on a 4-point Likert scale
Yan (2015, China)	Life satisfaction	Single item	Single item on a 4-point Likert scale
Yu (2019, China)	Quality of life	WHOQOL	World Health Organization Quality of Life Instruments
Zhang (2019, China)	Quality of life	WHOQOL-BREF	World Health Organization Quality of Life Instruments - Bref
Zhang (2019, China)	Quality of life	WHOQOL-BREF	World Health Organization Quality of Life Instruments - Bref
Zhang (2020, USA)	Life satisfaction	SWLS	5-item satisfaction with life scale
Zhang (2017, China)	Life satisfaction	LSI-A	20-item Life Satisfaction Index-A
Zhang (2017, China)	Life satisfaction	SWLS	5-item satisfaction with life scale
Zhang (2017, China)	Well-being	MIL	8-item Meaning in life
