# Supplementary material for: Neighborhood Attributes and Well-Being Among Older Adults in Urban Areas: A Mixed-Methods Systematic Review
Source: Res Aging. 2021 Apr 28;44(5-6):351–68. doi: 10.1177/0164027521999980 (PMC9039320; doi:10.1177/0164027521999980)
Supplement: Supplemental Material, sj-pdf-4-roa-10.1177_0164027521999980 - Neighborhood Attributes and Well-Being Among Older Adults in Urban Areas: A Mixed-Methods Systematic Review [file sj-pdf-4-roa-10.1177_0164027521999980.pdf]

Findings related to variables in studies

Studies	Well-being outcome	Physical environment						Service environment				Social environment					
		Cleanliness	Density and urban fabric	Natural areas	Overall evaluation	Overall neighbourhood environment	Walk. and public space	Health care	Local services	Social support	Transit	Age homogeneity	Deprivation	Ethnic diversity	Overall evaluation	Security	Sense of community
Au, 2020	Life satisfaction																
Chapman, 1983																	
Feng, 2018																	
Oswald, 2011																	
Park, 2017																	
Smith, 1995																	
Sugiyama, 2006																	
Xie, 2018																	
Yan, 2014																	
Yan, 2015																	
Zhang, 2020																	
Zhang, 2017 (USA)																	
Toma, 2015	Life satisfaction & Well-being																
Barresi, 1983	Well-being																
Chang, 2020																	
Cramm, 2013																	
Cramm, 2014																	
Cramm, 2015																	
Curl, 2019																	
Engel, 2016																	
Gao, 2017																	
He, 2020																	
Liu, 2017																	
Nieboer, 2018																	
Zhang, 2017 (China)	Well-being & Quality of life																
Curl, 2015	Quality of life																
Lane, 2020																	
Mottus, 2012																	
Paiva, 2019																	
Tiraphat, 2017																	
Ward Thompson, 2014																	

Yu, 2019																	
Zhang, 2019 (Hong Kong)																	
Zhang, 2019 (China)																	
Coleman, 2015	Qualitative methods																
Grant, 2007																	
Keene, 2013																	
Ottoni, 2016																	
Finlay, 2018																	
	**TOTAL**	**3**	**9**	**6**	**4**	**1**	**15**	**7**	**15**	**5**	**13**	**5**	**5**	**1**	**2**	**7**	**24**

Positive effect on SWB

Ambiguous/variable relationship with SWB

Negative effect on SWB

No effect
